# T1 mapping and speckle tracking echocardiography for the assessment of early mechanical dysfunction in transfusion-dependent β-thalassemia with normal T2*

**DOI:** 10.1016/j.jocmr.2026.102690

**Published:** 2026-01-16

**Authors:** Federico Marchini, Michele Malagù, Federica Frascaro, Elena Marchetti, Laura Rotondo, Maria Mele, Elisabetta Tonet, Rita Pavasini, Matteo Serenelli, Alberto Cossu, Serena Chiarello, Filomena Longo, Martina Culcasi, Olga Sofritti, Victoria Delgado, Gianluca Campo, Matteo Bertini

**Affiliations:** a Cardiology Unit, Azienda Ospedaliero Universitaria di Ferrara, Ferrara, Italy; b Division of Radiology, Azienda Ospedaliero Universitaria di Ferrara, Ferrara, Italy; c Thalassemia and Hemoglobinopathies Unit, S. Anna University Hospital, Ferrara, Italy; d UOC Ricerca e Innovazione, Azienda Ospedaliero-Universitaria di Ferrara, Ferrara, Italy; e Cardiovascular Imaging Unit, Department of Cardiology, Hospital Universitari Germans Trias i Pujol, Heart Institute, Badalona, Barcelona, Spain

**Keywords:** β-thalassemia, T1 mapping, Cardiovascular magnetic resonance, Speckle tracking echocardiography, Myocardial work

## Abstract

**Background:**

A T2* ≤20 ms in cardiovascular magnetic resonance (CMR) sequences suggests the presence of iron overload cardiomyopathy in patients with transfusion-dependent β-thalassemia (TDT). However, there is still a gap in evidence regarding the independent role of T1 mapping in identifying early myocardial dysfunction. The aim of this study is to investigate the role of T1 mapping in identifying early cardiac mechanical dysfunction in TDT patients with normal T2* values.

**Methods:**

About 154 consecutive TDT patients with T2* >20 ms were enrolled and stratified by reduced (≤955 ms) or normal (>955 ms) T1 mapping values. CMR T1 mapping and speckle tracking echocardiography (STE) indices were evaluated. The primary endpoint was the correlation between T1 mapping and STE indices. The secondary endpoint was the prevalence of cardiac mechanical dysfunction between patients with reduced or normal T1 mapping.

**Result:**

T1 mapping showed statistically significant correlations with global longitudinal strain (GLS, r = −0.19, p = 0.01), global work index (GWI, r = 0.15, p = 0.04), global constructive work (GCW, r = 0.18, p = 0.02), and peak atrial longitudinal strain (PALS, r = 0.2, p<0.01). The prevalence of cardiac mechanical dysfunction was low, without any difference between patients with reduced or normal T1 mapping.

**Conclusions:**

In TDT patients with normal T2*, T1 mapping demonstrated a weak but significant correlation with echocardiographic indices of cardiac mechanics. The prevalence of cardiac mechanical dysfunction was low without any difference between those with reduced or normal T1 mapping.

## Graphical abstract

**Figure:**
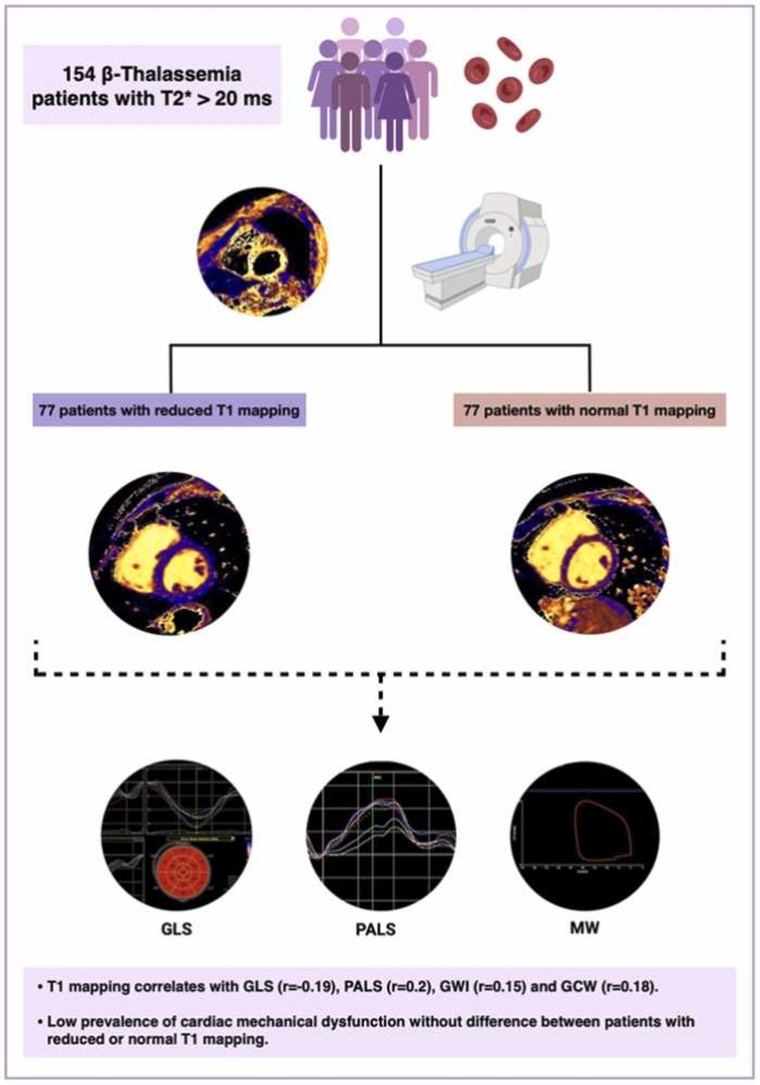
*GLS* global longitudinal strain, *PALS* peak atrial longitudinal strain, *GWI* global work index, *GCW* global constructive work

## 1 Background

Transfusion-dependent β-thalassemia (TDT) is a genetic blood disorder characterized by absent or reduced production of the β-chains of the hemoglobin [1], [2]. The introduction of early iron chelation therapy has been crucial in preventing life-threatening complications, such as heart failure and arrhythmias, contributing to a life expectancy almost comparable to that of the general population [3], [4]. Cardiovascular magnetic resonance (CMR) T2* mapping has led to a pivotal change in the natural history of the disease and nowadays has become a fundamental diagnostic tool for assessing myocardial iron overload (MIO) and guiding iron chelation therapy [5], [6], [7]. However, its sensitivity is limited in detecting mild or early MIO-related changes [8], [9], [10]. T1 mapping technique is a promising method to overcome this limitation and improve detection of MIO. Recent studies showed that T1 mapping is more sensitive for detecting patients at early stages of MIO which may not be identified using T2* alone [11], [12], [13]. Moreover, patients with higher levels of MIO show lower values of T1 mapping, carrying a significant risk of developing fatal cardiovascular complications [9].

Speckle tracking echocardiography (STE) with evaluation of global longitudinal strain (GLS) is able to evaluate cardiac contractile function, by overcoming the traditional drawbacks of routine left ventricular ejection fraction (LVEF) assessment [14]. More recently, myocardial work (MW), a further application of STE, has emerged as a valuable tool for assessing myocardial systolic function by combining both deformation parameters and loading conditions in its analysis [15]. Together, GLS and MW, allow for a comprehensive assessment of cardiac mechanics, capable of detecting early alterations in systolic function even before a reduction in LVEF occurs. While many studies have investigated the role of T2*, there is still a gap in evidence regarding the independent role of T1 mapping in identifying early mechanical dysfunction, especially in patients with normal T2*. The aim of this study was to investigate the role of T1 mapping in identify early cardiac mechanical dysfunction in TDT with normal T2*.

## 2 Methods

The present analysis has been derived from the “Atrial fibrillation in β-thalassemia *(*β-THAL)” study, which is a prospective, single-center, observational study conducted at the Azienda Ospedaliero-Universitaria di Ferrara, Italy. The study collected clinical, laboratory, electrocardiographic, and imaging characteristics in patients with TDT and was registered on ClinicalTrials.gov (NCT05508932). Study protocol was approved by the local Ethics Committee (Comitato Etico Indipendente di Area Vasta Emilia Centro). The study was conducted in accordance with the ethical principles of the Declaration of Helsinki and all patients signed informed consent. The corresponding author had full access to all the study data and takes responsibility for its integrity and the data analysis.

### 2.1 Study population

Consecutive TDT patients were enrolled. Inclusion criteria were confirmed TDT and age ≥18 years. Exclusion criteria were the state of pregnancy, inability to give informed consent, low quality of echocardiographic images that did not allow the execution of STE and absence of T1 mapping sequences on CMR examination. Patients underwent routine cardiological examination and CMR as the standard of care on an annual basis [16]. For the present analysis, only TDT patients with normal T2* (>20 ms) were included and stratified by reduced (≤955 ms) or normal (>955 ms) T1 mapping. Reduced T1 mapping has been defined as 2 standard deviations (SD) below the mean value of the reference range of our center [17].

### 2.2 Echocardiographic examination

A comprehensive echocardiographic, Doppler and Colour Doppler examination was performed using a GE Vivid E80, S60 or E9 echo scanner (GE Health Care, Milwaukee, Wisconsin) equipped with a 3.5 MHz transducer. Echocardiographic images were stored in digital format and analyzed using the EchoPAC software v. 202 (GE Health Care, Milwaukee, Wisconsin). Two expert trained cardiologists (F.M. and F.F.) performed all the echocardiographic measures, according with the American Society of Echocardiography/European Association of Cardiovascular Imaging guidelines [18], [19]. Image and Doppler acquisitions were obtained at held end-expiration. Non-invasive measurement of systolic and diastolic blood pressure has been performed during the exam by measurement in the arm using a sphygmomanometer. GLS, peak atrial longitudinal strain (PALS) and indices of MW (Global work index (GWI), global constructive work (GCW), global wasted work (GWW), and global work efficiency (GWE)) were obtained as previously reported [20]. Normal values were derived from published literature [20], [21], [22], [23]. The detailed echocardiography protocol is available on Supplementary Data.

### 2.3 CMR examination

**Fig. 1:**
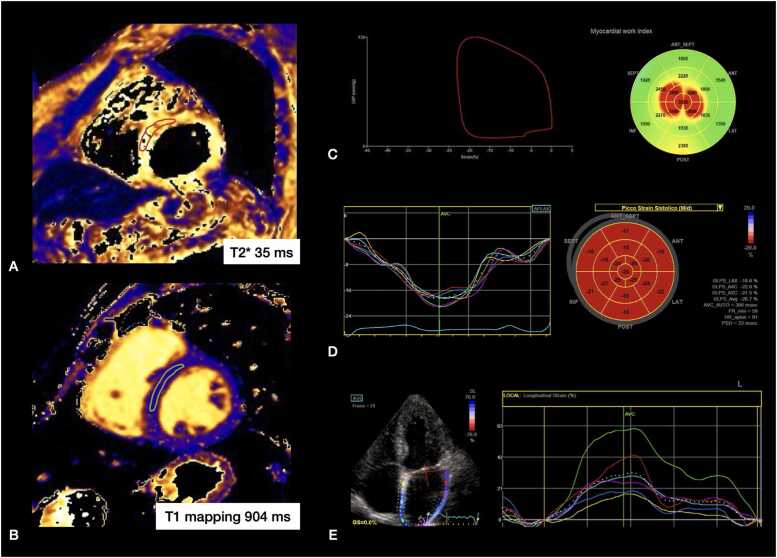
CMR and echo analysis, A TDT patient with normal T2* (A) but reduced T1 mapping (B). An ROI has been drawn in the mid-interventricular septum to obtain mapping values. Speckle tracking analysis in the same patient revealed normal values of MWI, GLS, and PALS (C, D, and F). *TDT transfusion-dependent* β-Thalassemia, *ROI* region of interest, *MWI* myocardial work index, *GLS* global longitudinal strain, *PALS* peak atrial longitudinal strain, *CMR* cardiovascular magnetic resonance

All CMR imaging procedures were performed with a 1.5T scanner (Siemens, Magnetom Aera, Siemens Healthineers, Erlangen, Germany) using a standard protocol. Post-processing analysis was performed using a commercially available software (IntelliSpace Portal, Philips Healthcare, Best, the Netherlands). All exams were acquired and analyzed by two expert radiologists (A.C., S.C.) and one expert cardiologist (F.M.). All images were obtained during breath holding at expiration. The cine images included the acquisition of one long-axis slice (two- and four-chamber) and a stack of short-axis (SAX) slices covering the entire LV using a balanced steady-state free precession sequence. Native T1 mapping was performed on three standard LV short-axis slices at basal, mid, and apical level using modified Look-Locker inversion recovery (MOLLI) sequences combined with motion correction (MOCO) and 3(3)3(3)5 scheme. Recovery rate of T1 relaxation was measured in a mid-ventricular SAX slice within the septal myocardium, as previously described and validated [24]. The mean T1 mapping value for our center was 1008 ms with 90% confidence interval (CI) 957–1060 ms. For MIO assessment, 3 SAX (basal, medium, and apical) slices were acquired using T2* dark blood gradient-echo multiecho sequences. A region of interest (ROI) was drawn in the interventricular septum and signal intensity was plotted against each echo time. T2* values were calculated from the resulting exponential decay curve and the truncation method was used to account for the plateau observed at the later echo times due to signal loss [25], [26]. For hepatic iron overload evaluation, a mid-hepatic slice was obtained. The detailed CMR protocol is available on the Supplementary Data. Fig. 1 shows representative CMR T2* and T1 calculation and echocardiographic evaluation of strain parameters and myocardial work.

### 2.4 Data collection

All data were prospectively collected using a dedicated electronic case record form (eCRF). Specialized personnel performed all procedures. The eCRFs were periodically monitored and verified using source data. The following data were collected: anthropometrics, cardiovascular (CV) risk factors, CV history and comorbidities, laboratory, last available transthoracic echocardiography, and CMR parameters. Data were collected from patients’ clinical records.

### 2.5 Study endpoints

The primary endpoint was the correlation between T1 mapping and echocardiographic indices of cardiac mechanics. The secondary endpoint was the prevalence of cardiac mechanical dysfunction, defined as the reduction in STE indices, between patients with reduced or normal T1 mapping.

### 2.6 Statistical analysis

Continuous data were tested for normal distribution with the Kolmogorov–Smirnov test. Normally distributed values were presented as mean ± standard deviation (SD) and compared to a t-test. Otherwise, median, interquartile range (IQR), and Mann–Whitney U test were applied. Categorical variables were summarized in terms of counts and percentages and were compared by using the two-sided Chi-square or Fisher’s exact test. The strength and direction of the relationship between T1 mapping and indices of cardiac mechanics were evaluated with Pearson's correlation. Univariate linear regression models were used to calculate linear regression coefficient and corresponding 95% confidence intervals (CIs) evaluating the relationship between each index of cardiac mechanics and T1 mapping. Significant variable at the 10% level (p-value <0.1) in the univariate analyses were included in a multivariate model. Clinical confounders (age, arterial hypertension, diabetes mellitus, and atrial fibrillation) were added to the multivariate models [27], [28]. The intra- and inter-observer agreements between operators were tested with the Bland Altman scatter plot analysis and intra-class correlation coefficients on the first 10 echocardiograms and CMRs were analyzed.

No previous study evaluated the correlation between T1 mapping and indices of cardiac mechanics in TDT patients with normal T2*. Based on available data, the correlation between T1 mapping and T2* in the 20–30 ms range is estimated to be approximately 0.5 [29], while the correlation between T2* and GLS is around 0.5–0.6 [30], [31]. Assuming a linear relationship between these parameters, we calculated that a minimum of 123 patients was required to obtain a correlation coefficient of −0.25 between T1 mapping and GLS, with 80% power and an alpha error of 0.05 (see Supplementary Data for detail).

## 3 Results

Between August 2022 and January 2025, 276 patients suffering from hemoglobinopathies were evaluated. Five patients with drepanocitosis and three patients with non-transfusion-dependent β-thalassemia were excluded. Fifteen patients showed T2* values ≤20 ms and, therefore, were excluded. Ninety-nine patients did not have an appropriate CMR exam (due to claustrophobia or absence of T1 mapping sequences) or had echocardiographic images of insufficient quality to perform strain analysis and MW and were also excluded. A comparison between baseline and echo characteristics between the study population and excluded patients is available on Supplementary Data, Table 1s.

**Fig. 2:**
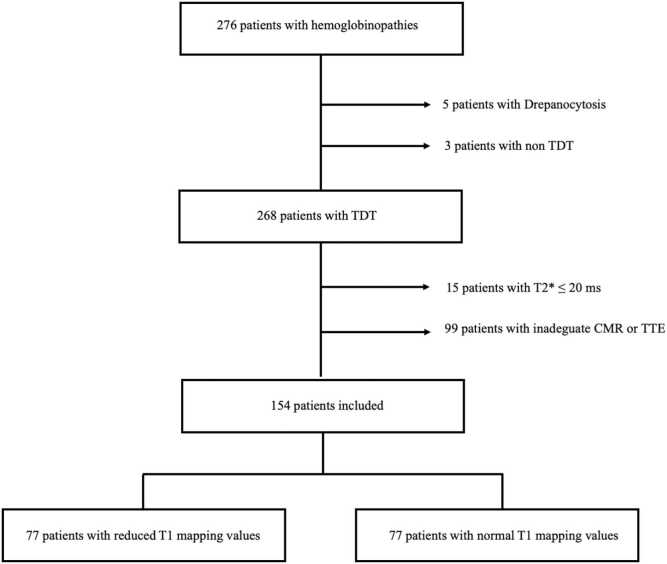
Flow chart of the study protocol. *TDT* transfusion-dependent β-Thalassemia, *CMR* cardiovascular magnetic resonance, *TTE* transthoracic echocardiography

Finally, 154 patients were included in the present analysis (Fig. 2), and among them, 77 patients (50%) had reduced T1 mapping values. Overall, the median age was 50 years (IQR 45–54), and the proportion of males was 49%. No differences were found in the clinical characteristics between patients with reduced or normal T1 mapping. Regarding the CMR parameters, patients with reduced T1 mapping had significantly lower values of T2* (37 vs 40 ms, p<0.001). Four patients with reduced T1 mapping had a right ventricular ejection fraction (RVEF) ≤ 50%. No differences were observed in the remaining CMR parameters. Baseline characteristics of the patients are summarized in Table 1.

**Table 1:** Baseline characteristic of the patients

	Total	Reduced T1 mapping (≤955 ms)	Normal T1 Mapping (>955 ms)	p-value
	N = 154	N = 77	N = 77	
*Clinical characteristics*				
Age, y	50 (45–54)	51 (46–54)	50 (44–54)	0.39
Male sex	76 (49%)	39 (51%)	37 (48%)	0.75
BMI, kg/m^2^	22.0 (20.7–24.5)	21.6 (20.2–23.6)	22.3 (21.1–25.1)	0.08
Systolic BP, mmHg	114 (13)	113 (13)	114 (14)	0.52
Diastolic BP, mmHg	70 (9)	69 (8)	71 (9)	0.11
Arterial hypertension, n%	12 (8%)	6 (8%)	6 (8%)	1.00
Dyslipidemia, n%	4 (3%)	4 (5%)	0 (0%)	0.04
Diabetes Mellitus, n%	28 (18%)	13 (17%)	15 (19%)	0.68
Hypothyroidism, n%	48 (31%)	24 (31%)	24 (31%)	1.00
Prior Stroke, n%	3 (2%)	2 (3%)	1 (1%)	1.00
Pulmonary hypertension, n%	7 (5%)	5 (6%)	2 (3%)	0.25
Prior splenectomy, n%	101 (66%)	52 (68%)	49 (64%)	0.61
COPD, n%	2 (1%)	1 (1%)	1 (1%)	1.00
CKD, n%	3 (2%)	1 (1%)	2 (3%)	0.57
History of HF, n%	4 (3%)	4 (5%)	0 (0%)	0.12
Atrial fibrillation, n%	20 (13%)	12 (16%)	8 (10%)	0.34
Pre-transfusion Hb, g/dl	10.0 (9.5–10.5)	10.1 (9.5–10.6)	10.0 (9.5–10.4)	0.07
Ferritin, ng/mL	526 (302–850)	429 (280–782)	583 (326–853)	0.15
*Echo characteristics*				
LV EDV indexed, mL/m^2^	57 (50–66)	55 (48–68)	58 (50–64)	0.87
LVEF, %	60 (59–65)	60 (59–65)	60 (59–65)	0.77
TAPSE, cm	2 (2–3)	2 (2–3)	2 (2–3)	0.81
PAPs, mmHg	25 (22–30)	26 (23–30)	25 (22–30)	0.68
RV EDA indexed, mL/m^2^	11 (10–13)	11 (10–13)	12 (10–13)	0.62
GLS, %	−21 (3)	−20 (3)	−21 (3)	0.22
GCW, mmHg%	2141 (396)	2089 (382)	2194 (405)	0.10
GWI, mmHg%	1931 (360)	1896 (352)	1966 (366)	0.23
GWW, mmHg%	58 (40–94)	58 (40–96)	64 (41–91)	0.79
GWE, %	96 (95–97)	96 (94–98)	97 (95–97)	0.89
PALS, %	36 (13)	34 (14)	37 (12)	0.19
*CMR characteristics*				
LV EDV indexed, mL/m^2^	84 (75–98)	84 (72–102)	85 (76–95)	0.98
LVEF > 50%, %	153 (99%)	76 (99%)	77 (100%)	1.00
RV EDV indexed, mL/m^2^	81 (70–97)	82 (65–102)	81 (71–94)	0.91
RVEF > 50%, %	150 (97%)	73 (95)	77 (100)	0.04
LV mass indexed, g/m^2^	48 (40–58)	46 (40–56)	50 (42–59)	0.31
T1 mapping, ms	954 (48)	915 (30)	993 (28)	<0.001
Cardiac T2*, ms	38 (35–41)	37 (34–40)	40 (37–45)	<0.001
Hepatic T2*, ms	9 (4–19)	9 (4–20)	9 (4–18)	0.42

*BMI* body mass index, *BP* blood pressure, *COPD* chronic obstructive pulmonary disease, *CKD* chronic kidney disease, *HF* heart failure, *Hb* hemoglobin, *LV* left ventricle, *EDV* end-diastolic volume, *LVEF* left ventricular ejection fraction, *TAPSE* tricuspid annular systolic excursion, *PAPs* systolic pulmonary artery pressure, *RV* right ventricle, *EDA* end-diastolic area, *GLS* global longitudinal strain, *GCW* global constructive work, *GWI* global work index, *GWW* global wasted work, *GWE* global work efficiency, *PALS* peak atrial longitudinal strain, *RVEF* right ventricular ejection fraction. Data are numbers (%) of cases, means ± standard deviation, or medians (interquartile range)

### 3.1 Echocardiographic characteristics

**Fig. 3:**
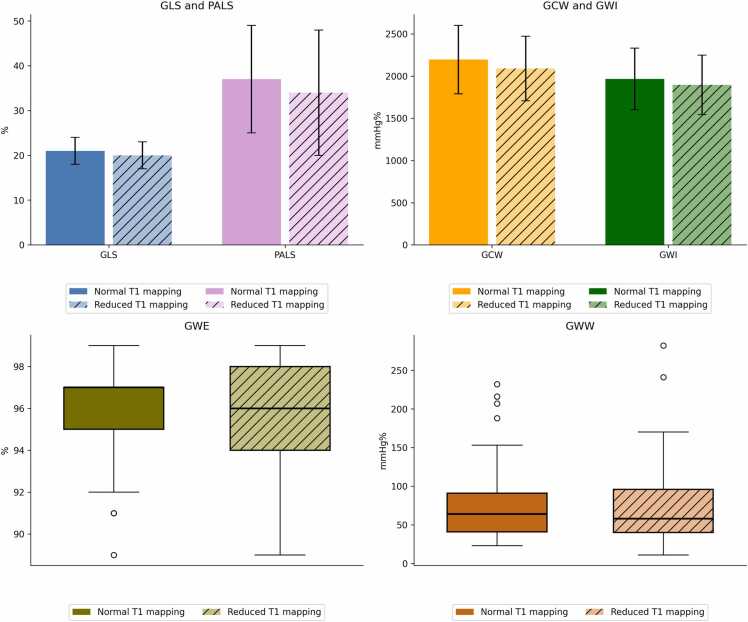
STE indices in the study groups. *GLS* global longitudinal strain, *GCW* global constructive work, *GWI* global work index, *GWW* global wasted work, *GWE* global work efficiency, *PALS* peak atrial longitudinal strain

In the overall population, the median LVEF was 60% while median left ventricular end-diastolic volume indexed was 57 mL/m^2^. Mean GLS was −20.3% ± 3 in patients with reduced T1 mapping and −21% ± 3 in patients with normal T1 mapping (p = 0.22). No significant difference was observed in MW indices between patients with reduced or normal T1 mapping. Finally, mean PALS was 34% ± 14 in patients with reduced T1 mapping and 37% ± 12 in patients with normal T1 mapping (p = 0.19) (Table 1 and Fig. 3).

### 3.2 Primary endpoint

**Fig. 4:**
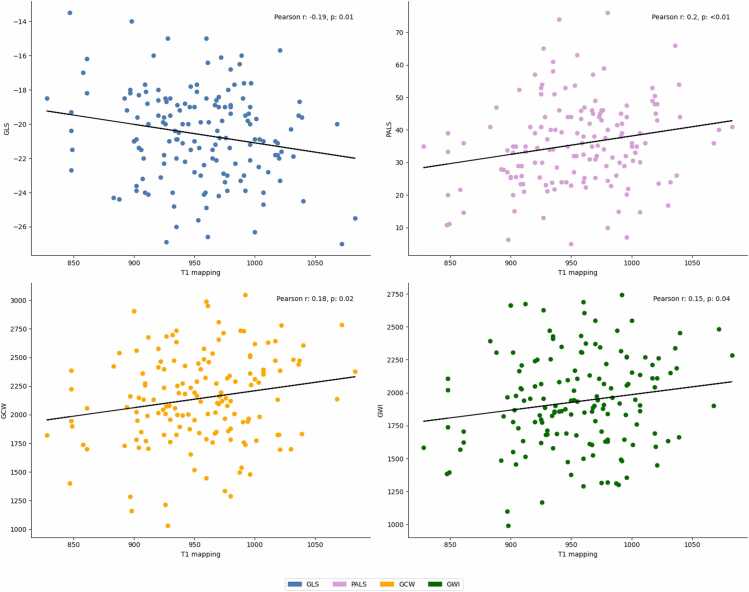
Correlation between T1 mapping and STE indices. *GLS* global longitudinal strain, *PALS* peak atrial longitudinal strain, *GCW* global constructive work, *GWI* global work index

T1 mapping showed statistically significant correlations with GLS (r = −0.19, p = 0.01), GWI (r = 0.15, p = 0.04), GCW (r = 0.18, p = 0.02), and PALS (r = 0.2, p = 0.009) (Table 2, Fig. 4). Linear regression analysis confirmed the linear associations between T1 mapping and GLS (β −0.01; 95% CI −0.01 to −0.002; p = 0.01), GCW (β 1.47; 95% CI 0.19–2.76, p = 0.02) and PALS (β 0.05; 95% CI 0.013–0.09, p = 0.01). After multivariate adjustment, T1 mapping showed an independent linear association with GLS, GWI, GCW, and PALS (Table 3).

**Table 2:** Correlation between T1 mapping/T2* and indices of cardiac mechanics

	*T1 mapping*		T2*	
*Variables*	Correlation coefficient	p value	Correlation coefficient	p value
*GLS*	−0.19	0.01	−0.17	0.02
*GWI*	0.15	0.04	0.14	0.06
*GCW*	0.18	0.02	0.16	0.04
*GWW*	−0.04	0.61	−0.08	0.27
*GWE*	0.14	0.07	0.18	0.02
*PALS*	0.2	0.009	0.11	0.15

*GLS* global longitudinal strain, *GCW* global constructive work, *GWI* global work index, *GWW* global wasted work, *GWE* global work efficiency, *PALS* peak atrial longitudinal strain

**Table 3:** Linear regression analysis between T1 mapping and indices of cardiac mechanics

*T1 mapping*				
*Variables*	Unadjusted β (95% CI)	p value	Adjusted β (95% CI)	p value
*GLS*	−0.01 (−0.01 to −0.002)	0.01	-0.01 (−0.12 to −0.003)	0.006
*GWI*	1.17 (0.001–2.35)	0.05	1.48 (0.32–2.63)	0.01
*GCW*	1.47 (0.19–2.76)	0.02	1.81 (0.55–3.08)	0.005
*GWW*	−0.03 (−0.19 to 0.11)	0.61		
*GWE*	0.006 (−0.001 to 0.13)	0.07	0.005 (−0.001 to 0.01)	0.08
*PALS*	0.05 (0.013–0.09)	0.01	0.04 (0.005–0.08)	0.02


T2*				

*Variables*	Unadjusted β (95% CI)	p value	Adjusted β (95% CI)	p value

*GLS*	−0.089 (−0.16 to −0.01)	0.02	−0.09 (−0.17 to −0.1)	0.01
*GWI*	9.63 (−0.60 to 19.86)	0.06	10.01 (−0.18 to 20.22)	0.05
*GCW*	11.45 (0.21–22.69)	0.04	11.43 (0.23–22.64)	0.04
*GWW*	−0.74 (−2.08 to 0.59)	0.27		
*GWE*	0.06 (0.01–0.12)	0.02	0.08 (0.02–0.13)	0.007
*PALS*	0.27 (−0.10 to 0.65)	0.15		

*GLS* global longitudinal strain, *GCW* global constructive work, *GWI* global work index, *GWW* global wasted work, *GWE* global work efficiency, *PALS* peak atrial longitudinal strain.

GLS, GCW, GWI, and GWE were adjusted for age, diabetes mellitus, and arterial hypertension.

PALS was adjusted for diabetes mellitus, atrial fibrillation, and arterial hypertension.

Moreover, T1 mapping showed a statistically significant correlation with hepatic T2* (r = −0.2, p = 0.01) and, when reduced, with serum ferritin (r = −0.2, p = 0.04).

Similarly, T2* revealed a statistically significant correlation with GLS (r = −0.17, p = 0.02), GCW (r = 0.16, p = 0.04), and GWE (r = 0.18, p = 0.02), and statistically significant linear associations with GLS (β −0.089; 95% CI −0.16 to −0.01; p = 0.02), GCW (β 11.45; 95% CI 0.21–22.69; p = 0.04), and GWE (β 0.06; 95% CI 0.01–0.12; p = 0.02), in both univariate analysis and after multivariate adjustment (Table 2 and Table 3).

### 3.3 Secondary endpoint

Reduced GLS values were observed in 5/77 patients (6%) with reduced T1 mapping and in 7/77 patients (9%) with normal T1 mapping (p = 0.55). Similarly, no significant differences were found in the proportion of patients with reduced GCW (8% in both groups [6/77], p = 1.00), GWI (4% [3/77] vs 1% [1/77], p = 0.31), GWW (1% [1/77] vs 0% [0/77], p = 0.32), GWE (0% [0/77] vs 1% [1/77], p = 0.32), or PALS (17% [13/77] vs 12% [9/77], p = 0.36) between patients with reduced or normal T1 mapping (Table 4).

**Table 4:** Proportion of patients with reduced STE indices in the study groups

	Total	Reduced T1 mapping (≤955 ms)	Normal T1 Mapping (>955 ms)	p-value
	N = 154	N = 77	N = 77	
GLS, n%	12 (8%)	5 (6%)	7 (9%)	0.55
GCW, n%	12 (8%)	6 (8%)	6 (8%)	1.00
GWI, n%	4 (3%)	3 (4%)	1 (1%)	0.31
GWW, n%	1 (1%)	1 (1%)	0 (0%)	0.32
GWE, n%	1 (1%)	0 (0%)	1 (1%)	0.32
PALS, n%	22 (14%)	13 (17%)	9 (12%)	0.36

*GLS* global longitudinal strain, *GCW* global constructive work, *GWI* global work index, *GWW* global wasted work, *GWE* global work efficiency, *PALS* peak atrial longitudinal strain. Data are numbers (%) of cases.

### 3.4 Intra-observer and inter-observer variability

Analysis for the first 10 patients was repeated 2 weeks after the initial analysis and no significant intra-observer variability was observed (GLS intra-class correlation (ICC): 0.98; GCW ICC: 0.99; GWI ICC: 0.98; GWW ICC: 0.97; GWE ICC: 0.85; PALS ICC: 0.92; T1 mapping ICC: 0.93). Measurements were also repeated by the second observer, showing excellent inter-observer reproducibility (GLS ICC: 0.92; GCW ICC: 0.98; GWI ICC: 0.95; GWW ICC: 0.99; GWE ICC: 0.92; PALS ICC: 0.98; T1 mapping ICC: 0.85) (Supplementary Data, Table 2s, Figs. 2s and 3s).

## 4 Discussion

To the best of our knowledge, this is the first study to evaluate the role of T1 mapping on cardiac performance through a comprehensive assessment of STE indices of cardiac mechanics in TDT patients with normal T2*. The main findings of the present study are the following:

1. T1 mapping demonstrated a statistically significant correlation with GLS, PALS, and MW, even though the correlation was weak, and an independent linear association remained after adjusting for clinical confounders.
2. The prevalence of cardiac mechanical dysfunction was low in both study groups without any statistically significant difference.

Cardiac magnetic resonance T2* has significantly changed the natural history of beta-thalassemia through the non-invasive assessment of cardiac iron deposition and long-term monitoring of therapeutic response [5], [6], [7]. T2* has proven to be a strong predictor of both heart failure and arrhythmic-related events, with even higher diagnostic performance than serum ferritin and liver iron deposition [32], [33], [34]. For decades, and still currently, T2* has been the most widely used and studied parameter for diagnosing and monitoring iron overload; however, its sensitivity in identifying mild iron deposition is limited, mainly due to susceptibility artifacts [8], [9], [10]. Moreover, the use of iron chelation therapy has led to an increasing number of patients with mild iron overload [35]. This raises the question whether T2* still represents a valid diagnostic and prognostic tool in these patients. More recently, native T1 mapping has emerged as a new diagnostic sequence for evaluation of iron deposition and new evidence strongly supports that it could be complementary to T2*. In the study of Torlasco et al., low T1 values were found in all patients with T2* <20 ms (r2 = 0.92, p<0.001) and in most of the patients in the T2* range 20–30 ms (r2 = 0.48, p<0.001), meaning that not only T1 and T2* are concordant, but also that T1 is able to identify MIO missed by T2* [29]. In the study of Selim et al., T1 mapping detected 2 patients with high MIO not assessed by T2*. Moreover, the authors found a statistically correlation between T1 values and the liver iron content, age, age at first transfusion, age of splenectomy, and serum ferritin value [13].

In the modern age of iron chelation therapy, it is not uncommon to observe TDT patients with T2* values >20 ms but with reduced T1 mapping. The meaning of this finding remains unknown, but it carries important consequences for patients in daily clinical practice. In the same way, it is not uncommon for patients with normal T2* values to develop clinically significant cardiac comorbidities, for which mild iron overload may be an undetected mechanism [36]. GLS and MW have shown to be reliable tools to detect myocardial impairment with higher sensibility and less dependent loading conditions than LVEF, allowing a more detailed insight of cardiac mechanics[14]. The correlation between myocardial dysfunction and MIO has been assessed in several studies. The majority of them found a significant correlation between GLS and T2* and proposed several cut-offs of GLS to predict iron overload [30], [31], [37]. Only a few studies assessed the correlation between strain parameters and T1 mapping, mainly showing negative results. Ojha et al. showed that only CMR-derived global radial strain displayed a significant correlation with T2* (r = 0.24, p = 0.02), while T1 mapping did not show any correlation with strain indices [38]. See et al. found no associations between T1 mapping or T2* with indices of ventricular deformation [39].

The aim of our study was to address the pivotal question whether T1 mapping can provide additional information for clinical management in TDT patients with a good chelation status according to T2* values. We found that almost all STE parameters of deformation and myocardial work displayed a linear association with T1 mapping, meaning that even a mild MIO correlates with changes in mechanical parameters. Notably, our study is, to our knowledge, the first to report a statistically significant correlation between native T1 values and most of the STE indices in TDT patients, as previous investigations have failed to demonstrate such an association. While the observed correlation was weak, this finding is consistent with the mild degree of iron accumulation and low prevalence of mechanical dysfunction in these patients. However, the most clinically important message is that when T2* is normal, the prevalence of cardiac mechanical dysfunction is low without any difference between patients with reduced or normal T1 mapping. Still, the low prevalence may entail a risk of underpowering. This finding carries fundamental implications for clinical practice: in fact, 1 of 2 TDT patients with normal T2* has reduced values of T1 mapping and, nowadays, the implication of this mild MIO was unknown. The present findings gave the first evidence to solve the uncertainty surrounding the interpretation of this discrepancy: among patients with normal T2*, the prevalence of cardiac mechanical dysfunction is low and T1 mapping does not provide an incremental benefit in identifying patients with reduced STE indices.

The second important message of this work is about the central role of T2*: in our study, not only patients with normal T2* had normal mean values of all indices of STE cardiac mechanics, but there was also a correlation between T2* values and STE indices. This finding remarked the important message that T2* between 21 and 40 ms does not rule out iron overload, since a mild iron accumulation may be present, but it remains a valuable tool to discriminate MIO-related dysfunction. Similarly, normal T1 mapping could not exclude mild iron accumulation either; however, when T2* exceeds 20 ms, the prevalence of cardiac mechanical dysfunction remains low regardless of T1 mapping values. These results further reinforce the role of T2* as the principal CMR sequence for early cardiac risk assessment in TDT patients, with T1 mapping conferring only marginal additional diagnostic value.

## 5 Limitations

The present investigation has several limitations that should be considered. First, data were collected from a single center and may not be representative of the entire population of TDT patients. Second, as the present data are cross-sectional, the absence of clinical outcomes and longitudinal follow-up precludes assessment of whether reduced T1 mapping predicts future cardiac dysfunction. Prospective studies with systematic follow-up are needed to confirm these findings. Third, the correlation coefficient found between T1 mapping and GLS was −0.19, meaning that the study might be slightly underpowered for the primary endpoint. However, since the lower critical r was −0.17, a statistically significant correlation was nevertheless observed. Additionally, due to the low prevalence of cardiac mechanical dysfunction, the type II error remains a concern for the secondary endpoint as well. Fourth, 99 of the eligible patients were excluded due to inadequate quality of echo imaging, introducing potential selection bias. To address this limitation, we compared baseline characteristics between included and excluded patients, finding no statistically significant differences (Supplementary Data, Table 1s). Fifth, data on previous MIO were not assessed, potentially missing the long-term effects on cardiac structure and function after myocardial iron levels have been normalized through chelation therapy. Sixth, we assessed the relationship between CMR T1 mapping and echocardiographic parameters of cardiac mechanics, which may introduce significant challenges in direct comparison and limit reproducibility. Emerging CMR feature tracking techniques are expected to enable a more direct and reproducible comparison of myocardial mechanics indices in future studies. Finally, we did not record data about clinical events, which might occur independently of the presence of cardiac dysfunction detected by STE, further highlighting the need for longitudinal studies.

## 6 Conclusions

In TDT patients with normal T2*, T1 mapping was correlated with myocardial deformation parameters and most of the indices of MW. The prevalence of cardiac mechanical dysfunction was low without any difference between those with reduced or normal T1 mapping.

## Funding

The authors declare that no funds, grants, or other support were received during the preparation of this manuscript.

## Author contributions

**Federico Marchini:** Writing – original draft, Validation, Methodology, Investigation, Data curation, Conceptualization. **Michele Malagù:** Supervision, Investigation, Conceptualization. **Federica Frascaro:** Writing – original draft, Validation, Investigation. **Elena Marchetti:** Investigation. **Laura Rotondo:** Investigation. **Maria Mele:** Investigation. **Elisabetta Tonet:** Supervision. **Rita Pavasini:** Supervision. **Matteo Serenelli:** Investigation. **Alberto Cossu:** Supervision, Investigation. **Serena Chiarello:** Investigation. **Filomena Longo:** Supervision. **Martina Culcasi:** Investigation. **Olga Sofritti:** Investigation. **Victoria Delgado:** Writing – review & editing, Supervision. **Gianluca Campo:** Writing – review & editing, Supervision. **Matteo Bertini:** Writing – review & editing, Supervision.

## Declaration of competing interests

The authors declare that they have no known competing financial interests or personal relationships that could have appeared to influence the work reported in this paper.

## Data Availability

All data relevant to the study are included in the article or uploaded as supporting information.

## References

[1] Musallam K.M., Cappellini M.D., Viprakasit V., Kattamis A., Rivella S., Taher A.T. (2021). Revisiting the non‐transfusion‐dependent (NTDT) vs. transfusion‐dependent (TDT) thalassemia classification 10 years later. Am J Hematol.

[2] Ali S., Mumtaz S., Shakir H.A., Khan M., Tahir H.M., Mumtaz S. (2021). Current status of beta‐thalassemia and its treatment strategies. Mol Genet Genom Med.

[3] Gordeuk V.R., Bacon B.R., Brittenham G.M. (1987). Iron overload: causes and consequences. Annu Rev Nutr.

[4] Ozment C.P., Turi J.L. (2009). Iron overload following red blood cell transfusion and its impact on disease severity. Biochim Et Biophys Acta (BBA) Gen Subj.

[5] Ramazzotti A., Pepe A., Positano V., Rossi G., De Marchi D., Brizi M.G. (2009). Multicenter validation of the magnetic resonance T2* technique for segmental and global quantification of myocardial iron. J Magn Reson Imaging.

[6] Carpenter J.-P., He T., Kirk P., Roughton M., Anderson L.J., de Noronha S.V. (2011). On T2* magnetic resonance and cardiac iron. Circulation.

[7] Pepe A., Meloni A., Rossi G., Midiri M., Missere M., Valeri G. (2018). Prediction of cardiac complications for thalassemia major in the widespread cardiac magnetic resonance era: a prospective multicentre study by a multi-parametric approach. Eur Heart J Cardiovasc Imaging.

[8] Alam M.H., Auger D., Smith G.C., He T., Vassiliou V., Baksi A.J. (2015). T1 at 1.5T and 3T compared with conventional T2* at 1.5T for cardiac siderosis. J Cardiovasc Magn Reson.

[9] Krittayaphong R., Zhang S., Saiviroonporn P., Viprakasit V., Tanapibunpon P., Komoltri C. (2017). Detection of cardiac iron overload with native magnetic resonance T1 and T2 mapping in patients with thalassemia. Int J Cardiol.

[10] Roghi A., Poggiali E., Pedrotti P., Milazzo A., Quattrocchi G., Cassinerio E. (2015). Myocardial and hepatic iron overload assessment by region-based and pixel-wise T2* mapping analysis. J Comput Assist Tomogr.

[11] Meloni A., Martini N., Positano V., De Luca A., Pistoia L., Sbragi S. (2021). Myocardial iron overload by cardiovascular magnetic resonance native segmental T1 mapping: a sensitive approach that correlates with cardiac complications. J Cardiovasc Magn Reson.

[12] Ibrahim H.R., Ahmed A.T. (2023). Role of cardiac magnetic resonance T1 mapping in comparison to T2* for cardiac iron overload assessment in transfusion-dependent thalassemia major patients. Egypt J Radiol Nucl Med.

[13] Selim O.M.H.Z., Ibrahim A.S.A.H., Aly N.H., Hegazy S.N.A., Ebeid F.S.E. (2024). Early detection of myocardial iron overload in patients with β-thalassemia major using cardiac magnetic resonance T1 mapping. Magn Reson Imaging.

[14] Geyer H., Caracciolo G., Abe H., Wilansky S., Carerj S., Gentile F. (2010). Assessment of myocardial mechanics using speckle tracking echocardiography: fundamentals and clinical applications. J Am Soc Echocardiogr.

[15] Moya A., Buytaert D., Penicka M., Bartunek J., Vanderheyden M. (2023). State-of-the-art: noninvasive assessment of left ventricular function through myocardial work. J Am Soc Echocardiogr.

[16] Pepe A., Pistoia L., Gamberini M.R., Cuccia L., Lisi R., Cecinati V. (2022). National networking in rare diseases and reduction of cardiac burden in thalassemia major. Eur Heart J.

[17] Popescu I.A., Werys K., Zhang Q., Puchta H., Hann E., Lukaschuk E. (2021). Standardization of T1-mapping in cardiovascular magnetic resonance using clustered structuring for benchmarking normal ranges. Int J Cardiol.

[18] Lang R.M., Badano L.P., Mor-Avi V., Afilalo J., Armstrong A., Ernande L. (2015). Recommendations for cardiac chamber quantification by echocardiography in adults: an update from the American Society of Echocardiography and the European Association of Cardiovascular Imaging. Eur Heart J Cardiovasc Imaging.

[19] Mitchell C., Rahko P.S., Blauwet L.A., Canaday B., Finstuen J.A., Foster M.C. (2019). Guidelines for performing a comprehensive transthoracic echocardiographic examination in adults: recommendations from the American Society of Echocardiography. J Am Soc Echocardiogr.

[20] Manganaro R., Marchetta S., Dulgheru R., Ilardi F., Sugimoto T., Robinet S. (2019). Echocardiographic reference ranges for normal non-invasive myocardial work indices: results from the EACVI NORRE study. Eur Heart J Cardiovasc Imaging.

[21] Voigt J.-U., Pedrizzetti G., Lysyansky P., Marwick T.H., Houle H., Baumann R. (2015). Definitions for a common standard for 2D speckle tracking echocardiography: consensus document of the EACVI/ASE/Industry Task Force to standardize deformation imaging. Eur Heart J Cardiovasc Imaging.

[22] Nyberg J., Jakobsen E.O., Østvik A., Holte E., Stølen S., Lovstakken L. (2023). Echocardiographic reference ranges of global longitudinal strain for all cardiac chambers using guideline-directed dedicated views. JACC Cardiovasc Imaging.

[23] Nielsen A.B., Skaarup K.G., Hauser R., Johansen N.D., Lassen M.C.H., Jensen G.B. (2021). Normal values and reference ranges for left atrial strain by speckle-tracking echocardiography: the Copenhagen City Heart Study. Eur Heart J Cardiovasc Imaging.

[24] Messroghli D.R., Moon J.C., Ferreira V.M., Grosse-Wortmann L., He T., Kellman P. (2016). Clinical recommendations for cardiovascular magnetic resonance mapping of T1, T2, T2* and extracellular volume: a consensus statement by the Society for Cardiovascular Magnetic Resonance (SCMR) endorsed by the European Association for Cardiovascular Imaging (EACVI). J Cardiovasc Magn Reson.

[25] Schulz-Menger J., Bluemke D.A., Bremerich J., Flamm S.D., Fogel M.A., Friedrich M.G. (2020). Standardized image interpretation and post-processing in cardiovascular magnetic resonance - 2020 update. J Cardiovasc Magn Reson.

[26] He T., Gatehouse P.D., Smith G.C., Mohiaddin R.H., Pennell D.J., Firmin D.N. (2008). Myocardial *T* measurements in iron‐overloaded thalassemia: an in vivo study to investigate optimal methods of quantification. Magn Reson Med.

[27] Claus P., Omar A.M.S., Pedrizzetti G., Sengupta P.P., Nagel E. (2015). Tissue tracking technology for assessing cardiac mechanics. JACC Cardiovasc Imaging.

[28] Voigt J.-U., Cvijic M. (2019). 2- and 3-dimensional myocardial strain in cardiac health and disease. JACC Cardiovasc Imaging.

[29] Torlasco C., Cassinerio E., Roghi A., Faini A., Capecchi M., Abdel-Gadir A. (2018). Role of T1 mapping as a complementary tool to T2* for non-invasive cardiac iron overload assessment. PLoS One.

[30] Pizzino F., Meloni A., Terrizzi A., Casini T., Spasiano A., Cosmi C. (2018). Detection of myocardial iron overload by two-dimensional speckle tracking in patients with beta-thalassaemia major: a combined echocardiographic and T2* segmental CMR study. Int J Cardiovasc Imaging.

[31] Garceau P., Nguyen E.T., Carasso S., Ross H., Pendergrast J., Moravsky G. (2011). Quantification of myocardial iron deposition by two-dimensional speckle tracking in patients with -thalassaemia major and Blackfan-Diamond anaemia. Heart.

[32] Kirk P., Roughton M., Porter J.B., Walker J.M., Tanner M.A., Patel J. (2009). Cardiac T2* magnetic resonance for prediction of cardiac complications in thalassemia major. Circulation.

[33] Marchini F., Fiorio A., Sirugo P., Gamberini M.R., Mari E., Bertini M. (2022). Heavy metal! A case of severe iron overload and supraventricular arrhythmias in a thalassemia major patient. G Ital Cardiol.

[34] Malagù M., Marchini F., Fiorio A., Sirugo P., Clò S., Mari E. (2022). Atrial fibrillation in β-thalassemia: overview of mechanism, significance and clinical management. Biology.

[35] Alavi S., Ebadi M., Ghazizadeh F., Arzanian M.T., Shamsian B., Abdollah Gorji F. (2014). Efficacy and safety of deferasirox in β-Thalassemia major patients in Iran: a prospective study from a single referral center in Iran. Pedia Hematol Oncol.

[36] Malagù M., Tonet E., Orazio G., Longo F., De Raffele M., Sirugo P. (2024). Association between epicardial adipose tissue and atrial fibrillation in patients with transfusion-dependent β-Thalassemia. J Clin Med.

[37] Ari M.E., Ekici F., Çetin İ.İ., Tavil E.B., Yaralı N., Işık P. (2017). Assessment of left ventricular functions and myocardial iron load with tissue Doppler and speckle tracking echocardiography and T2* MRI in patients with β‐thalassemia major. Echocardiography.

[38] Ojha V., Ganga K.P., Seth T., Roy A., Naik N., Jagia P. (2021). Role of CMR feature-tracking derived left ventricular strain in predicting myocardial iron overload and assessing myocardial contractile dysfunction in patients with thalassemia major. Eur Radiol.

[39] See W.-S., So E.K., Hwang G.Y.-Y., Chin L., Ip L., Lam W.W. (2022). Native cardiac magnetic resonance T1 mapping and cardiac mechanics as assessed by speckle tracking echocardiography in patients with beta-thalassaemia major. IJC Heart Vasc.

