# Supplementary material for: T1 mapping and speckle tracking echocardiography for the assessment of early mechanical dysfunction in transfusion-dependent β-thalassemia with normal T2*
Source: J Cardiovasc Magn Reson. 2026 Jan 16;28(1):102690. doi: 10.1016/j.jocmr.2026.102690 (PMC13237548; doi:10.1016/j.jocmr.2026.102690)
Supplement: Supplementary file 1 — Supplementary material [file mmc1.docx]

**Supplementary Data**

**T1 mapping and speckle tracking echocardiography for the assessment of early mechanical dysfunction in transfusion-dependent β-thalassemia with normal T2***

- **Detailed echocardiography protocol page 2**
- **Detailed CMR parameters page 4**
- **Sample size calculation page 5**
- **Table 1s page 6**
- **Table 2s page 7**
- **Table 3s page 8**
- **Figure 2s page 9**
- **Figure 3s page 10**
- **Detailed echocardiography protocol**

**Acquisition**

Left ventricle (LV) end-diastolic and end-systolic volumes were assessed from 4-chamber (CH) view and 2-CH view using the biplane Simpson rule. Biplane left ventricular ejection fraction (LVEF) was calculated from the standard equation. Cardiac valve regurgitations were graded conform to current EACVI guidelines^1^. The tricuspid annular plane systolic excursion (TAPSE) was evaluated on the M-mode trace. Right ventricle end-diastolic area was calculated at end-diastole on apical 4-CH view. For the 2D speckle tracking echocardiography (STE) image acquisition, sector size and depth were adjusted to achieve optimal visualization of the whole myocardium of the left ventricle in the three apical views, achieving a frame rate between 40 and 80 fps^2^.

**Strain and myocardial work post-processing**

End-systole was assessed by the aortic valve closure in the apical long-axis view. The regions of interest (ROIs) were outlined along myocardial borders at end-systole in the apical views. Longitudinal myocardial strain was calculated throughout the myocardium for each LV apical view and reported in a polar plot map (bull’s eye) using a colour-coded representation. A 17-segment model of the left ventricle was used for evaluation of longitudinal myocardial strain^2^. PALS was assessed in 4 and 2-CH views outlining the atrial borders^3^ and the mean value was reported.

Myocardial work was measured from pressure-strain loop (PSL). Valvular events times were set by PW Doppler. The following MW indices were calculated: global work index (area under the loop), global constructive work, global wasted work and global work efficency^4^.

**Normal values used for STE indices**

GLS: < -17.2%^5^

PALS: > 23%^6^

Global work index: > 1292 mmHg%^4^

Global constructive work: > 1582 mmHg%^4^

Global wasted work: < 254 mmHg%^4^

Global work efficiency: > 90%^4^

**Detailed CMR parameters**

**bSSFP:** repetition time (TR) / echo time (TE): 30/1.2 ms; Field of view (FOV) read/FOV phase: 380 mm / 84%; flip angle (FA): 56°; GRAPPA factor of 2; voxel size: 1.2 x 1.2 x 8 mm; slice thickness: 8 mm; distant factor: 20%

**MOLLI:** TR / TE: 377/1.2 ms; FOV read/FOV phase: 320 mm / 85%; FA: 35°; GRAPPA factor of 2; voxel size: 1.3 x 1.3 x 8 mm; slice thickness: 8 mm; distant factor: 150%

**Black blood T2*:** TR 657 ms; TE: 10 echos every 2.26 ms starting from 2 ms; FOV read/FOV phase: 400 mm / 82%; FA: 25°; GRAPPA factor of 2; voxel size: 1.1 x 1.1 x 8 mm; slice thickness: 8 mm; distant factor: 150%

**Sample size calculation**

**Exact** - Correlation: Bivariate normal model

**Options:** exact distribution

**Analysis:** A priori: Compute required sample size

**Input:** Tail(s) = Two

Correlation ρ H1 = 0,25

α err prob = 0,05

Power (1-β err prob) = 0,8

Correlation ρ H0 =

**Output:** Lower critical r = -0,1771325

Upper critical r = 0,1771325

Total sample size = 123

Actual power = 0,8022355

**Figure 1s.** Power Curve

**Table 1s.** Baselines characteristics between the study population and excluded patients

|  | **Total** | **Excluded patients** | **Study population** | **p-value** |  |
| --- | --- | --- | --- | --- | --- |
|  | N=253 | N=99 | N=154 |  | |
| **Clinical Characteristics** |  |  |  |  | |
| Age, yrs | 50 (45-55) | 52 (46-57) | 50 (45-54) | 0.087 | |
| Male sex | 118 (47%) | 42 (42%) | 76 (49%) | 0.28 | |
| BMI, kg/m2 | 22.2 (20.8-24.5) | 22.7 (20.9-24.4) | 22.0 (20.7-24.5) | 0.39 | |
| Arterial hypertension, n% | 25 (10%) | 13 (13%) | 12 ( 8%) | 0.16 | |
| Dyslipidemia, n% | 5 ( 2%) | 1 ( 1%) | 4 ( 3%) | 0.65 | |
| Diabetes Mellitus, n% | 54 (21%) | 26 (26%) | 28 (18%) | 0.13 | |
| Hypothyroidism, n% | 74 (29%) | 26 (26%) | 48 (31%) | 0.40 | |
| Prior Stroke, n% | 5 ( 2%) | 2 ( 2%) | 3 ( 2%) | 1.00 | |
| Pulmonary hypertension, n% | 12 ( 5%) | 5 ( 5%) | 7 ( 5%) | 0.85 | |
| Prior splenectomy, n% | 156 (62%) | 55 (56%) | 101 (66%) | 0.11 | |
| COPD, n% | 5 ( 2%) | 3 ( 3%) | 2 ( 1%) | 0.38 | |
| CKD, n% | 7 ( 3%) | 4 ( 4%) | 3 ( 2%) | 0.44 | |
| History of HF, n% | 7 ( 3%) | 3 ( 3%) | 4 ( 3%) | 1.00 | |
| Atrial fibrillation, n% | 42 (17%) | 22 (22%) | 20 (13%) | 0.054 | |
| Pre-transfusion Hb, g/dl | 10 (9.5-10.7) | 10.2 (9.5-11) | 10 (9.5-10.5) | 0.11 | |
| Ferritin, ng/ml | 531 (322-841) | 547 (359-836) | 526 (302-850) | 0.69 | |
|  |  |  |  |  | |
| **Echo characteristics** |  |  |  |  | |
| LV EDV indexed, ml/m2 | 56 (48-65) | 54 (46-64) | 57 (50-66) | 0.15 | |
| LVEF, % | 60 (59-65) | 61 (60-65) | 60 (59-65) | 0.97 | |
| TAPSE, cm | 2 (2-3) | 2 (2-3) | 2 (2-3) | 0.47 | |
| PAPs, mmHg | 27 (23-32) | 29 (25-33) | 25 (22-30) | 0.13 | |
| RV EDA indexed, ml/m2 | 11 (10-13) | 11 (11-15) | 11 (10-13) | 0.054 | |

*BMI: body mass index, BP: blood pressure; COPD: chronic obstructive pulmonary disease; CKD: chronic kidney disease; HF: heart failure; Hb: hemoglobin; LV: left ventricle; EDV: end-diastolic volume; LVEF: left ventricular ejection fraction; TAPSE: tricuspid annular systolic excursion; PAPs: systolic pulmonary artery pressure; RV: right ventricle; EDA: end-diastolic area*

**Table 2s.** Linear regression analysis between T1 mapping and indices of cardiac mechanics after adjustment for T2*

| T1 mapping | | |  |
| --- | --- | --- | --- |
| Variables | Adjusted β (95% CI) | P value | |
|  |  |  | |
| GLS | -0.009 (-0.01 - 0.001) | 0.08 | |
| GWI | 1.21 (-0.16 – 2.59) | 0.08 | |
| GCW | 1.56 (0.06 – 3.07) | 0.04 | |
| GWW | 0.05 (-0.12 – 0.23) | 0.55 | |
| GWE | 0.001 (-0.006 – 0.009) | 0.75 | |
| PALS | 0.03 (-0.006 – 0.08) | 0.09 | |

*GLS: global longitudinal strain; GCW: global constructive work; GWI: global work index; GWW: global wasted work; GWE: global work efficiency; PALS: peak atrial longitudinal strain.*

GLS, GCW, GWI, GWW and GWE were adjusted for age, diabetes mellitus, arterial hypertension and T2*

PALS was adjusted for diabetes mellitus, atrial fibrillation, arterial hypertension and T2*

**Inter-observer and intra-observer variability**

**Table 3s.** Reproducibility of data using intraclass correlation coefficients

| Variables | Inter-observer reproducibility | Intra-observer reproducibility |  |
| --- | --- | --- | --- |
|  |  |  | |
| GLS | 0.92 (0.76 – 0.97) | 0.98 (0.94 – 0.99) | |
| GCW | 0.98 (0.96 – 0.99) | 0.99 (0.98 – 0.99) | |
| GWI | 0.95 (0.86 – 0.98) | 0.99 (0.97 – 0.99) | |
| GWW | 0.99 (0.97 – 0.99) | 0.97 (0.92 – 0.99) | |
| GWE | 0.92 (0.75 – 0.97) | 0.85 (0.57 – 0.95) | |
| PALS | 0.98 (0.95 – 0.99) | 0.92 (0.76 – 0.98) | |
| T1 mapping | 0.85 (0.59 – 0.96) | 0.93 (0.77 – 0.98) | |

Data are represented using intraclass correlation coefficients (95% CI)

GLS: global longitudinal strain; GCW: global constructive work; GWI: global work index; GWW: global wasted work; GWE: global work efficiency; PALS: peak atrial longitudinal strain.

**Figure 2s.** Bland Altman plots for inter-observer variability


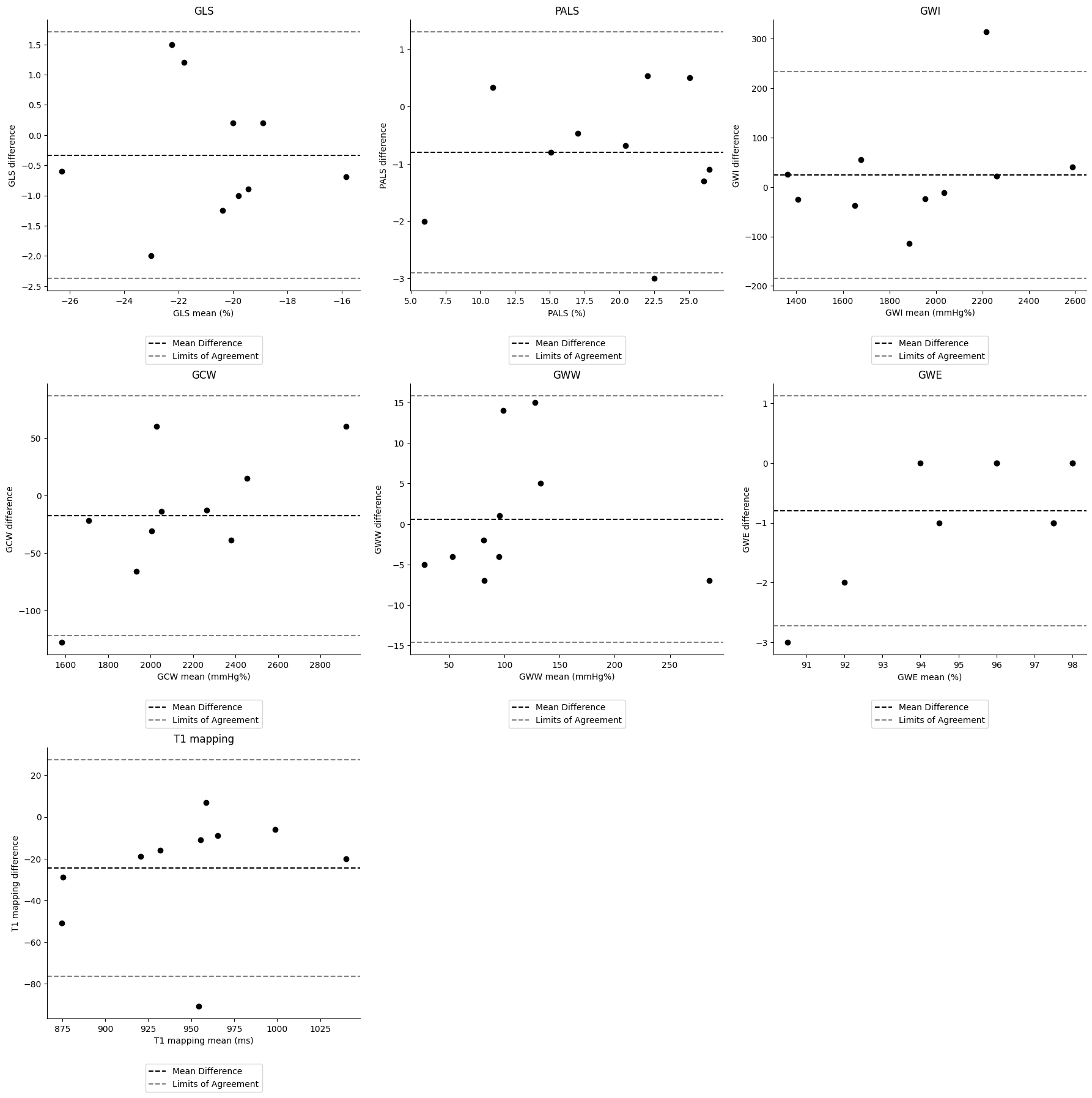


The Bland–Altman analysis for assessing inter-observer variability of indices of cardiac mechanics and T1 mapping. Dotted lines represent bias and 95% limits of agreement for measurements performed in 20 patients.

**Figure 3s.** Bland Altman plots for intra-observer variability


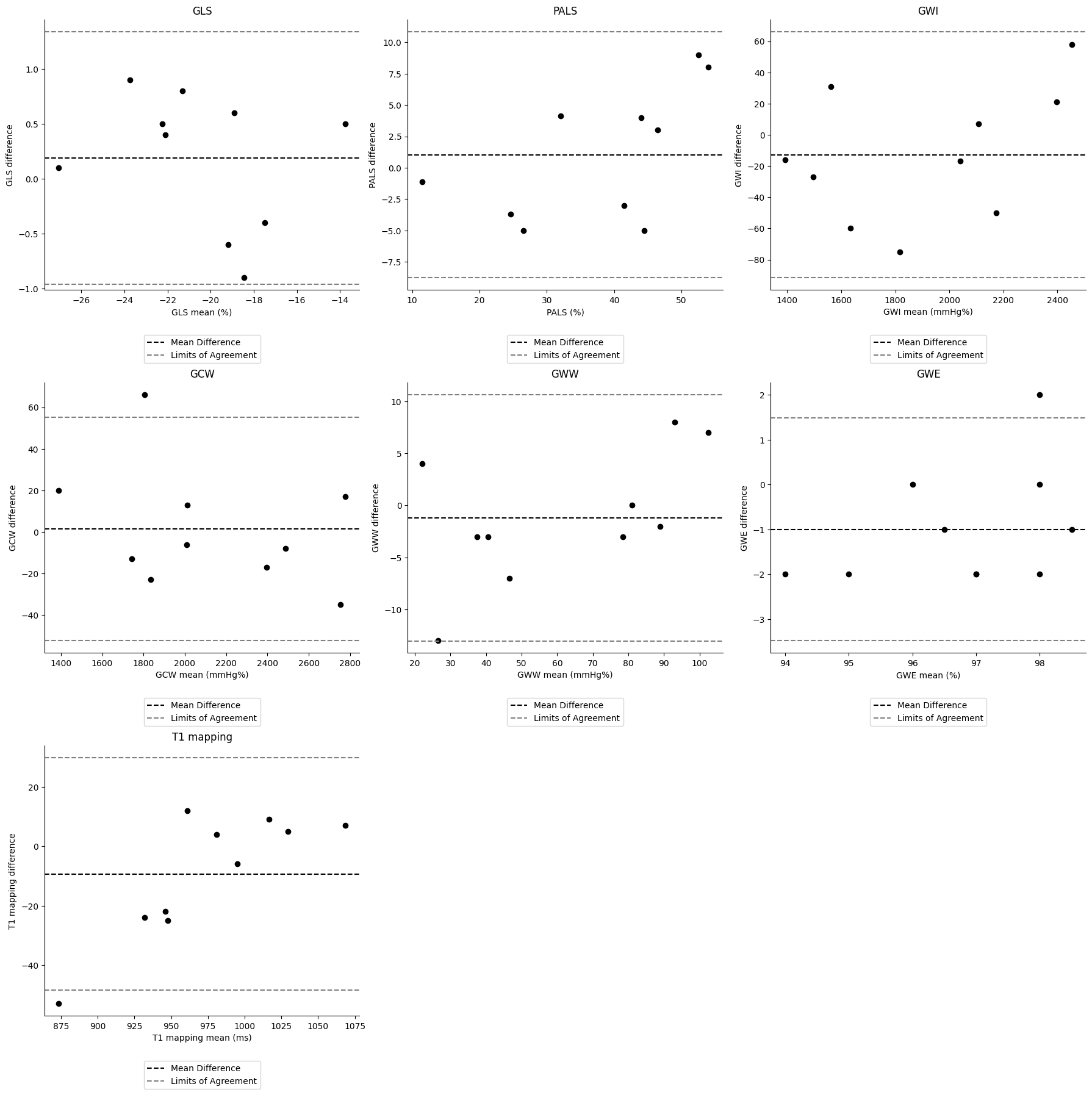


The Bland–Altman analysis for assessing intra-observer variability of indices of cardiac mechanics and T1 mapping. Dotted lines represent bias and 95% limits of agreement for measurements performed in 20 patients.

**REFERENCES**

1. Lancellotti P, Pibarot P, Chambers J, Canna G La, Pepi M, Dulgheru R, Dweck M, Delgado V, Garbi M, Vannan MA, Montaigne D, Badano L, Maurovich-Horvat P, Pontone G, Vahanian A, Donal E, Cosyns B. Multi-modality imaging assessment of native valvular regurgitation: an EACVI and ESC council of valvular heart disease position paper. *Eur Heart J Cardiovasc Imaging* 2022;**23**:e171–e232.

2. Voigt J-U, Pedrizzetti G, Lysyansky P, Marwick TH, Houle H, Baumann R, Pedri S, Ito Y, Abe Y, Metz S, Song JH, Hamilton J, Sengupta PP, Kolias TJ, d’Hooge J, Aurigemma GP, Thomas JD, Badano LP. Definitions for a common standard for 2D speckle tracking echocardiography: consensus document of the EACVI/ASE/Industry Task Force to standardize deformation imaging. *Eur Heart J Cardiovasc Imaging* 2015;**16**:1–11.

3. Pathan F, D’Elia N, Nolan MT, Marwick TH, Negishi K. Normal Ranges of Left Atrial Strain by Speckle-Tracking Echocardiography: A Systematic Review and Meta-Analysis. *Journal of the American Society of Echocardiography* 2017;**30**:59-70.e8.

4. Manganaro R, Marchetta S, Dulgheru R, Ilardi F, Sugimoto T, Robinet S, Cimino S, Go YY, Bernard A, Kacharava G, Athanassopoulos GD, Barone D, Baroni M, Cardim N, Hagendorff A, Hristova K, López-Fernández T, la Morena G de, Popescu BA, Penicka M, Ozyigit T, Rodrigo Carbonero JD, Veire N van de, Bardeleben RS Von, Vinereanu D, Zamorano JL, Rosca M, Calin A, Moonen M, Magne J, Cosyns B, Galli E, Donal E, Carerj S, Zito C, Santoro C, Galderisi M, Badano LP, Lang RM, Oury C, Lancellotti P. Echocardiographic reference ranges for normal non-invasive myocardial work indices: results from the EACVI NORRE study. *Eur Heart J Cardiovasc Imaging* 2019;**20**:582–590.

5. Sugimoto T, Dulgheru R, Bernard A, Ilardi F, Contu L, Addetia K, Caballero L, Akhaladze N, Athanassopoulos GD, Barone D, Baroni M, Cardim N, Hagendorff A, Hristova K, Lopez T, la Morena G de, Popescu BA, Moonen M, Penicka M, Ozyigit T, Rodrigo Carbonero JD, Veire N van de, Bardeleben RS von, Vinereanu D, Zamorano JL, Go YY, Rosca M, Calin A, Magne J, Cosyns B, Marchetta S, Donal E, Habib G, Galderisi M, Badano LP, Lang RM, Lancellotti P. Echocardiographic reference ranges for normal left ventricular 2D strain: results from the EACVI NORRE study. *Eur Heart J Cardiovasc Imaging* 2017;**18**:833–840.

6. Nielsen AB, Skaarup KG, Hauser R, Johansen ND, Lassen MCH, Jensen GB, Schnohr P, Møgelvang R, Biering-Sørensen T. Normal values and reference ranges for left atrial strain by speckle-tracking echocardiography: the Copenhagen City Heart Study. *Eur Heart J Cardiovasc Imaging* 2021;**23**:42–51.
